# A Tolerance Study of Turmeric Extract in Healthy Adult Cats

**DOI:** 10.3390/ani16091355

**Published:** 2026-04-28

**Authors:** Emilie Raynaud, Melody Raasch, William Sanders, Denise Mitchell, Jeremy Laxalde, Vincent Biourge, Claudie Venet, Todd Cohen

**Affiliations:** Royal Canin Research Center, 650 Avenue de la Petite Camargue, 30470 Aimargues, France; emilie.raynaud@royalcanin.com (E.R.);

**Keywords:** cat, curcuminoid, safety, tolerance, turmeric

## Abstract

Turmeric has a long history of use as a colorant and flavoring agent. Turmeric extract (TE) is a feed additive containing at least 90% total curcuminoids, comprising mainly curcumin, desmethoxycurcumin and bisdemethoxycurcumin. The published antioxidant effects of TE in humans have sparked interest and feeding studies in companion animals. Studies describing the feeding of TE to cats are scarce and do not provide adequate toxicology data; regulatory approval is required to allow use of TE as a nutritional antioxidant in pet food. The current study describes a safety test of TE in cats. Control cats were fed a standard extruded dry diet whilst two groups of test cats were fed the same diet supplemented with two different levels of TE for four months. Physical examination, body weight, body condition score, food intake, fecal score, monitoring of adverse effects (vomiting, diarrhea, clinical signs), complete blood count, and blood biochemistry (particularly liver enzymes) were used to monitor toxicity signs. The lack of statistically significant effects of clinical or toxicological concern concludes that feeding TE to cats at a dietary level providing up to 1040 ppm total curcuminoids is safe. This allows future application of this ingredient in cat food as a nutritional antioxidant.

## Simple Summary

Turmeric is a bright yellow spice found in the rhizome (underground stem) of the *Curcuma longa* plant. For centuries, turmeric has been used as a cooking spice and colorant; its antioxidant properties have been exploited for the preservation of food and have led to its use in dietary supplements. More recently, studies have shown some benefits of turmeric extract (TE) in cats and dogs but safety data at higher doses are lacking. The current study reports a safety test of higher TE doses in cats. Two levels of TE were applied to dry cat food and fed daily to healthy cats for four months. A control group of cats fed the same diet without TE was used for comparison. Cats were regularly monitored for signs of toxicity, bodyweight, feces quality and general health to assess any adverse effects of TE. When compared with control cats, the cats fed TE showed no statistical differences in health concern in any of the measures tested. This study supports safe feeding of TE to cats daily for four months at a level in cat food delivering up to 1040 ppm total curcuminoids. This knowledge shows that TE can be safely added to cat food.

## 1. Introduction

Turmeric is the common name for the rhizome of *Curcuma longa*, an herbaceous perennial plant. The vibrant, deep yellow color and distinctive bitter, aromatic flavor of turmeric have popularized its use in foods and cooking. Under the designation E100, turmeric is approved in the European Union (EU) as a flavor and colorant additive. Turmeric is rich in curcuminoids, of which curcumin is the major constituent comprising around 75–80% of crude rhizome extract [1]. The other curcuminoids, desmethoxycurcumin and bisdemethoxycurcumin, make up around 15–20% and 3–5% respectively [1]. Together, curcumin, desmethoxycurcumin and bisdemethoxycurcumin are referred to as total curcuminoids. Curcuminoids are lipophilic, polyphenolic compounds with potent antioxidant activity, prompting the use of turmeric extract (TE), a concentrated and bioavailable form of curcuminoids, as a preservative in foods [2]. Reports of antioxidant-related benefits in humans have promoted the widespread inclusion of TE in dietary supplements [3,4,5,6].

The evidence for benefits of TE in humans, combined with an increasing interest in the use of evidence-based functional pet foods, opens a potential opportunity for the application of TE to companion animal diets. This has prompted a small number of short-term studies in which cats and dogs were fed diets containing TE. One such study tested the efficacy and safety in cats of a diet enriched with several ingredients including TE, providing 83 mg/1000 kcal curcuminoids (equivalent in this study to an intake of 4 mg/kg bodyweight (BW)/day), without associated adverse effects [7]. TE has also been tested in cats in combination with green-lipped mussel and blackcurrant leaf extract [8]. In this study, an oral dose of TE delivering 28.5 mg curcuminoids daily (4.75 mg/kg BW/day) for 10 weeks was not associated with any side effects. There are also published feeding studies in dogs describing the testing of curcumin or turmeric [9,10,11,12]. Whilst the outcomes of these studies were mixed in terms of efficacy, none of them reported adverse effects.

Low levels of TE are permitted in animal feeds as an alternative to synthetic colorants. The European Food Safety Authority (EFSA) has published a paper describing safe inclusion of TE in animal feed at a level of 15 mg/kg, with a maximum safe concentration set at 22 mg/kg for cats in a feed containing 88% dry matter [13]. This level is limited to use of TE as a sensory additive, which is defined as a substance added to animal feed or human food to improve or change its organoleptic properties. This dietary limit was calculated based on a 3 kg cat consuming 68 g of diet per day, which equates to an intake of 0.5 mg TE/kg BW/day. It should be noted that in the absence of suitable feeding studies in cats, this recommendation is extrapolated from a reproductive toxicity study in rats in which a No Observed Adverse Effect Level (NOAEL) of 250–320 mg/kg BW/day was reported [14]. Cats have an unusually low capacity for glucuronidation, which is an important metabolic step in the metabolism and subsequent excretion of curcuminoid metabolites [15]. This peculiarity increases the risk of turmeric toxicity in cats. For this reason, coupled with the lack of published relevant safety studies in cats, EFSA recommends a relatively low maximum dosage which takes into account intra- and interspecies uncertainty factors. In humans and mice, the feeding of diets containing high levels of TE, ranging from 2000 to 20,000 mg/kg, have been associated with changes in iron metabolism and the induction of iron deficiency anemia [16,17]. Cats have a unique hemoglobin structure which ultimately results in reduced intrinsic oxygen affinity and increased susceptibility to Heinz body formation, characterized by clumps of oxidized hemoglobin inside red blood cells. Increased numbers of Heinz bodies are an indicator of oxidative stress and represent a key screening marker in cat safety studies relating to test substances with which anemia has been previously associated.

Based on previous data showing an efficacious range of 250–600 ppm TE in cat diets [7], dietary levels higher than those permitted for the provision of color or flavor effects in animal feed are necessary to achieve nutritional benefits. The lack of data relating to the feeding of such levels to cats, and the potential association of TE with iron deficiency means there remains a need to establish evidence-based safe daily dose levels of TE in this species. The aim of this study was to test the safety of two dietary levels of TE in cats when fed daily for four months. Dietary concentrations of TE which delivered 660 (Diet A) and 1040 ppm (Diet B) total curcuminoids were selected based on an internal evaluation; this took into account published feeding studies in cats, official guidance on conducting toxicological studies, and toxicologist advice. Safety was evaluated via frequent monitoring of health status, general behavior, physical examination, BW, body condition score (BCS), food intake, feces score, adverse events, complete blood count with specific focus on markers of anemia, and blood chemistry. We hypothesized that feeding the two diets containing TE to cats daily for four months would not result in any clinically significant alterations in the above measures compared with cats fed the same diet containing no TE.

## 2. Materials and Methods

### 2.1. Ethical Approval

This study was approved by the Institutional Animal Care and Use Committee (IACUC) of Clinvet, South Africa (CG1671-CVSA24/420) and Royal Canin S.A.S., Aimargues, France (project code 131469) and was performed between October 2024 and March 2025 at Clinvet, South Africa. The research protocol complied with welfare standards set by AAALAC International and was conducted in accordance with the Veterinary International Cooperation on Harmonization Good Clinical Practice Guideline (VICH GL9, June 2000).

### 2.2. Powering of Study

A power analysis was performed to determine the appropriate number of cats for the study. The required sample size was estimated to statistically detect an effect size of 1.2, expressed as Cohen’s d. The sample size was calculated using an independent two-sided *t*-test with a power of 80% and an alpha risk of 0.1 to give a minimum sample size of 10 per group. This was increased by 50% to 15 animals per group to ensure maintenance of the minimum required number of participants in the event of study withdrawals.

### 2.3. Animals and Inclusion Criteria

The study population included 45 healthy, adult, domesticated shorthaired cats. Cats were purpose bred at the study facility, for research purposes. Cats were selected based on criteria as follows: bodyweight range of 2.5–6 kg, age of >1 year, good clinical health as confirmed by veterinarian examination, no symptoms of chronic digestive problems within three months of study initiation, absence of fleas or ticks, and feces scores of 2–3 as measured on a 9-point scale where 1: very crumbly stool; 1.5: hard and crumbly stool; 2: molded, hard stool; 2.5: molded, firm stool; 3: molded but soft stool; 3.5: unmolded stool, soft with form; 4: unmolded stool, soft without form; 4.5: diarrheal stool with consistency; 5: diarrheal stool without consistency. All cats were dewormed the week before study acclimation using Triworm-C (containing 20 mg Praziquantel and 230 mg Pyrantel per tablet), at a dose of one tablet per 4 kg BW. Cats wore electronic transponders with unique alphanumeric codes for identification purposes throughout the study. All cats remained in the facility after study completion.

### 2.4. Animal Husbandry

The study was conducted at Clinvet (Clinglobal Group, Bloemfontein, South Africa). Study cats were located in two outdoor animal units in which they were individually housed throughout the four month study (see Supplementary Table S1 for details of accomodation units). Heating was provided by means of an infrared heater/globe or by heating panel, dependent on the specific housing unit. Lighting was achieved by natural sunlight. Individual cages were labeled with the unique identification code of the cat, study number, group number and diet code. Cats were fed once daily and were provided with fresh drinking water ad libitum.

### 2.5. Study Design and Randomization

The study was a parallel, controlled, single-center, blinded tolerance trial, following a randomized block design. A four-month duration was recommended as the minimum for the tolerance trial as this period ensures at least one complete red blood cell (RBC) turnover cycle in cats, whose average RBC lifespan is approximately 70 days.

A total of 45 cats were ranked by decreasing day-7 bodyweight and blocked into 15 groups of 3 cats before random allocation to one of three groups (control, Diet A, Diet B) within block, using a random number generator in Microsoft Excel. Ascending order of animal ID was used to break ties. The resulting three groups were balanced for age and baseline bodyweight distribution. Sex and neuter status were similarly distributed across groups although the control group contained only males that were neutered whilst both TE groups contained an intact male each. Details of baseline characteristics of each group are shown in Table 1. Research personnel were blinded to treatment groups and diets whilst the sponsor representative and statistician were not.

All cats were acclimated for 14 days prior to the study start, during which they received the control diet daily. For the next 121 days, cats received their relevant diet according to study group.

### 2.6. Study Diets

All three study diets were based on a single batch of standard, non-commercial, dry, nutritionally complete and balanced diet for healthy adult cats. Diets were manufactured by Royal Canin^®^ SAS (Aimargues, France) and were formulated according to nutritional levels established by the Association of American Feed Control Officials (AAFCO) (Table 2). Control cats were given the standard (control) diet throughout the study period. The two test treatment groups received the standard diet supplemented with a curcuminoid-rich commercial TE (9024160 Curcuma Phospholipids, Meriva^®^, Indena, Milan, Italy), achieved by coating the kibble with TE in combination with fat. Additional proprietary palatants were applied to the test diets to ensure acceptance.

Diets A and B were designed to contain target doses of approximately 600 and 1200 ppm total curcuminoids respectively. Blinded analysis of total curcuminoid content in finished diets using an accredited and validated HPLC-UV-vis method performed by ECAM RICERT Laboratory (Gecchelina, Italy) confirmed actual total curcuminoid level of 660 ppm (Diet A) and 1040 ppm (Diet B) (Table 2 and Supplementary Table S2). Note that minor discrepancies (less than 15% of deviation) between target and analyzed doses are expected due to inherent process variability and limitations of the analytical method. Diets were isocaloric and were provided in plain bags, showing a coded name for blinded differentiation.

Stability of total curcuminoids in the kibble has been previously assessed over the 18-month shelf life of the kibble products at two doses of 250 and 275 ppm (unpublished data). The curcuminoids showed stability across 18 months, within acceptable limits (<20% decrease) from the initial T0 analysis performed straight after manufacture.

### 2.7. Routine Sample Collection and Analysis

Following a minimum of 12 h fasting, a blood sample of approximately 8 mL was collected by jugular venipuncture of each cat on days 0, 30, 62, 90 and 120. Of this, approximately 5 mL of whole blood was removed into a blood tube containing EDTA for complete blood count (CBC) analysis. The remaining 3 mL collected for biochemistry was allowed to stand for 30 min at room temperature and centrifuged at 1250 *g* for 10 min to separate the serum. CBC and biochemistry samples were analyzed by Clinomics (Bloemfontein, South Africa) according to standard manufacturer’s instructions using automated analyzers for animal blood: XN-1000V Hematology Analyzer (Sysmex, Kobe, Japan) and AU480 Chemistry Analyzer (Beckman Coulter, Tokyo, Japan).

### 2.8. Additional Sample Collection and Analysis

At day 120, due to investigation of an adverse event, all cats in the Diet B treatment group underwent additional blood sampling for analysis of prothrombin time (PT), partial thromboplastin time (PTT), and fibrinogen. These samples were analyzed within 24 h of collection at IDEXX Laboratories Pty Ltd. (Johannesburg, South Africa).

### 2.9. Observations and Examinations

Daily general health observations of the cats were conducted cageside from day-25 to 120. Abnormal signs were recorded and escalated to the veterinarian who examined and treated accordingly. Physical examination and body condition score (BCS) assessments were conducted on each cat at day-14 by the veterinarian and thereafter weekly for the duration of the study. The physical examination consisted of obtaining a meaningful history via reference to the animal history card, a thorough physical examination including environmental observations if applicable, and performing appropriate ancillary tests where necessary. BCS was determined on a 1*–*9 scale according to the descriptions shown in Supplementary Table S3 where scores of 1*–*3 are under ideal, 4*–*6 are ideal and 7*–*9 are over ideal.

### 2.10. Bodyweight Monitoring

Cats were weighed on a calibrated and verified electronic scale (ZEMIC-A12E-150 kg, model L6E3, Zemic Europe B.V., Breda, The Netherlands) on days-25, -14, and weekly thereafter until day 120. Weighing took place at approximately the same time of day and cats were weighed in the same order. Day 0 bodyweights were used as the reference point to detect decreases or increases in bodyweight during the study. Any individual weight increase of >5% from day 0 resulted in a reduction in rationing by 10% for that cat, with continued weight monitoring and ration adjustment as required. Animals that lost weight but continued to eat all of their ration remained in the study and had their ration increased by 10%, with continued weight monitoring. Animals that lost weight and did not complete their daily ration were removed from the study unless their weight loss was less than 5% and general wellbeing, water consumption and clinical signs were acceptable.

### 2.11. Food Consumption

Cats were fed to maintain BW; energy intakes were set individually according to neuter status and day-25 BW, using the following formula:

Metabolic energy requirement (MER) = 78–93 kcal BW^0.711^/day

where BW = bodyweight (kg) [18].

This equated to 36–79 g diet/day for intact cats and 31–66 g/day for neutered cats. Food consumption for each cat was recorded daily throughout the study. The daily ration was weighed prior to offering and any food remaining after 19 ± 1 h was weighed and recorded. Total daily food intakes were calculated by subtracting food remaining from food offered, allowing percentage of offered food consumed to be determined. To encourage appetence, cats were fasted for at least 4 h prior to food being offered.

### 2.12. Fecal Scoring

Feces were monitored daily from days-25 to 120 and collected from the litterbox for visual inspection. The feces were cut into four equally sized pieces with a spatula and the firmness, texture, structure, and moisture content of each quadrant estimated using a 9-point feces scoring scale, as follows: 1: very crumbly stool; 1.5: hard and crumbly stool; 2: molded, hard stool; 2.5: molded, firm stool; 3: molded but soft stool; 3.5: unmolded stool, soft with form; 4: unmolded stool, soft without form; 4.5: diarrheal stool with consistency; 5: diarrheal stool without consistency. The final feces score was determined by averaging the scores of the four quadrants.

### 2.13. Complete Blood Count (CBC) and Blood Biochemistry

Standard CBC and biochemistry parameters were measured for each cat at each blood sampling timepoint, with a particular focus on markers of anemia. Reference ranges for CBC and biochemistry parameters were taken from published values [19,20].

CBC parameters measured were as follows: basophils, eosinophils, hematocrit, hemoglobin, immature reticulocyte fraction, lymphocytes, mean cell hemoglobin (MCH), mean cell hemoglobin concentration (MCHC), mean cell volume (MCV), monocytes, segmented neutrophils, platelet count, red blood cell count (RBC), red cell distribution width, reticulocyte hemoglobin, reticulocytes, and white blood cell count (WBC). A blood smear was stained and evaluated microscopically for abnormal cell morphology, specifically focusing on the presence or absence of Heinz bodies.

The following biochemistry parameters were measured: alanine aminotransferase (ALT), albumin, alkaline phosphatase (ALP), amylase, aspartate aminotransferase (AST), calcium, chloride, cholesterol, creatine kinase, creatinine, direct bilirubin, gamma-glutamyl transferase (GGT), globulin, glucose, phosphorus, lactate dehydrogenase, magnesium, potassium, sodium, total bilirubin, total serum protein (TSP), and urea.

### 2.14. Statistical Analyses

SAS Version 9.4 (SAS Institute Inc., Cary, NC, USA) was used for all statistical calculations with procedures (e.g., PROC MIXED, PROC GLM, PROC FREQ) from the licensed SAS/STAT module. All testing was two-sided and conducted at the 10% significance level unless otherwise stated. The significance level (α) was set at 0.10 to enhance sensitivity for detecting potential adverse effects. This approach is consistent with regulatory toxicology practices and aligns with principles outlined in Food and Drug Administration (FDA) guidance (CVM GFI #226) for safety studies.

Descriptive statistics were applied to CBC and biochemical parameters, detailing change from baseline (day 0) to each sampling day. Within-group comparisons were made using ANOVA, with sample time as fixed effect and animal as random effect. Pairwise comparisons between each sampling day and day 0 were conducted using linear contrasts. Between-group comparisons of CBC and biochemistry data were analyzed using a mixed model for repeated measures which included, group, day and group × day interaction as fixed effects, the corresponding day 0 value as the covariate, day as the repeated measure, subject (cat) as the subject for repeated assessments, and randomization block as the random effect. Between-group comparisons were made by comparing each treated group to the control group. Different covariance structures (unstructured, compound symmetry, heterogeneous compound symmetry, first-order autoregressive, and heterogenous first-order autoregressive) were evaluated to model the correlation between repeated observations for the same animal. The structure with the lowest Akaike Information Criterion (AIC) was selected for the final model. The fixed effects in the model were assessed as follows: Step 1—if the group × day interaction was significant at α = 0.10, pairwise comparisons between each group and the control group within each day were performed using linear contrasts at an unadjusted α = 0.10. Step 2—if the interaction was not significant, the main effect of group was evaluated at α = 0.10. If significant, pairwise comparisons between each group and the control group were performed using linear contrasts at an unadjusted α = 0.10.

Bodyweights and change from day 0 were presented for each animal and summarized using descriptive statistics for each group by assessment day. BW of Day-25 was used for food offering (ration) calculations; the BW of Day 0 was used as the reference BW for comparison of an increase/decrease in BW during the study. Within-group comparisons were conducted as described for CBC and biochemical parameters. Between-group comparisons were also conducted similarly except that pre-treatment BW was not treated as a covariate. Percentage change in BW was calculated for each day and incidents of >5% absolute change identified as a binomial outcome of >5% or ≤5%. A BW variation of ≤5% was considered acceptable. The absolute % BW change was assessed at each day as acceptable or not and the mean proportion (probability) of acceptable events over the study period was calculated for each group using a generalized linear model (GLM) for binomial responses with a log link function. This model included group as a fixed effect. The analysis used a one-sided α = 0.10. For acceptable BW changes, the hypothesis tested for each group separately was whether the proportion of acceptable events was above the target of 80%.

Average weekly food consumption and fecal scores were calculated and summarized descriptively. Between-group analyses were performed similarly to those previously described, except that study week, rather than day, was used as the fixed factor along with the appropriate interactions. Pre-exposure values (days-6 to 0) were averaged to provide baseline covariates. Acceptable food consumption was defined per offering as consumption of ≥80% of food offered. Food consumption was assessed each day as acceptable or not acceptable and mean proportion (probability) of acceptable events over the study period calculated per group using a GLM for binomial responses with a log link function. This model included group as fixed effect and the analysis used a one-sided α = 0.10. For acceptable food consumption, the hypothesis tested, for each group separately, was whether the proportion of acceptable events was above the target of 80%. Acceptable feces scores were analyzed similarly by categorizing the fecal score each day. Acceptable fecal scores were defined as ≥1.5 and ≤3.5; the hypothesis tested for each group separately was whether the proportion of acceptable events was above a target of 75%.

BCS values were summarized descriptively and between-group analyses performed as described for CBC and biochemistry data.

Total numbers of adverse events (including total number of vomiting events) per animal were used to calculate group means. Treatment groups were then compared to control using a linear mixed model (LMM) with group as fixed effect and randomization block as random effect using a two-sided α = 0.10. All other measures or observations were recorded and listed descriptively.

Boxplots presented in the analysis include outliers as defined by the default SAS graphical procedures, based on the Tukey method (i.e., observations falling outside 1.5 times the interquartile range [IQR] from the first and third quartiles), and these are shown for transparency.

## 3. Results

### 3.1. Summary of Cats Completing Study

A total of 39/45 cats completed the study. Six cats were removed (three from control group, one from the Diet A group and two from the Diet B group) at various timepoints for the following reasons: continued bodyweight loss (n = 2 in control, n = 1 in the Diet B group), an adverse event involving appearance of skin lesions in the area of previous surgery (n = 1 in control group), inappetence for three consecutive days (n = 1 in the Diet A group) and a serious adverse event (n = 1 in the Diet B group). The serious adverse event involved a sudden, unexpected death of a cat on day 90. Complete necropsy was conducted alongside an analysis of the cat’s clinical history throughout the study. Histopathology of necropsy samples did not reveal a definitive cause of death; however, it was concluded that it was unlikely to be related to the trial given the lack of any definitive lesions deemed secondary to ingestion of the test diet. To investigate further, additional blood analyses of coagulation parameters, PT, PTT and fibrinogen were conducted on this cat, all cats in this treatment group at day 120, and on any cats in the facility which were blood-related to the deceased cat; all were found to be within normal ranges.

### 3.2. Bodyweight, Body Condition Score and Food Intake

There were significant differences in mean weekly BW between diet A and control group at weeks 8–14 and week 16 (Supplementary Table S4). Mean weekly BW was also significantly different for diet B versus controls for weeks 7 and 8 (Supplementary Table S4).

There were statistically significant (*p* < 0.10) differences in BW change from baseline between the diet A and control groups at day 7. For the diet B group, statistically significant differences in BW change from baseline were seen at days 56, 63, 77 and 91 compared with the control group. There were no other significant differences between treatment and control groups for this parameter at any other timepoint.

Within-group comparisons of acceptable changes in BW from baseline showed mean absolute percentage changes of <5% in all three groups; this ranged from 0.99% to 3.81% in the control group, 1.43% to 4.46% in the Diet A group and 0.97% to 3.99% in the Diet B group.

Mean day 0 body condition score (BCS) was 5.8 ± 0.86 (control group), 5.7 ± 0.82 (Diet A group) and 5.3 ± 0.88 (Diet B group) and by day 120 was 5.4 ± 1.56, 5.6 ± 0.74 and 5.4 ± 1.26 respectively. Tests for fixed effects were non-significant for the two-way group × day interaction and main effect of group.

All three diets were observed to be palatable. There were several instances of food adjustment (+/−10%) as follows: control group (n = 5) on a total of 14 occasions, Diet A group (n = 5) on 11 occasions, and Diet B group (n = 6) on 14 occasions. Weekly mean daily food intakes ranged from 42 to 54 g in control cats, 48–54 g in the Diet A group and 45–52 g in the Diet B group. See Supplementary Table S5 for mean daily food intakes by study week according to treatment group. A test for fixed effects on weekly food consumption showed the two-way interaction (group × day) to be significant (*p* < 0.0001) and the main effect of group non-significant (*p* = 0.4763). Pairwise group comparisons by week showed no significant differences in food intakes in either test group compared with control throughout the 120 day study period. Comparisons of mean weekly food consumption resulted in a statistically significant effect at week 1 only, whereby the Diet B group showed a significantly higher food intake compared with control (*p* = 0.036). There were no other significant differences between groups found at any other weekly timepoint of the study.

Acceptable food consumption, defined as consuming ≥80% of the food offered, was compared between control and test treatment groups. The lower 90% one-sided confidence interval (CI) for the proportion of acceptable events was 80.8% for the control group (*p* = 0.015) compared with 87.8% for the Diet A group (*p* < 0.001) and 78.6% for the Diet B group (*p* = 0.562), where significance denotes the proportion of offered food consumed is >80%. Overall, there were no consistent significant differences in food consumption associated with the TE supplemented diets compared with the control.

### 3.3. Fecal Scores

Group mean fecal scores across the 120-day study period showed no significant differences between Diet A and Diet B groups compared with control. Feces scores of ≥1.5 or ≤3.5 were present in 77.0% of feces for the control group, 82.4% in the Diet A group, and 79.9% in the Diet B group. A test for fixed effects was significant for the two-way group × day interaction (*p* < 0.0001) but not for the main effect of group (*p* = 0.3007). Pairwise group comparisons by week showed a significantly higher fecal score in the Diet A group versus control at weeks 2, 3, and 16, and a significantly lower fecal score in the Diet B group versus control at week 5, with no other significant effects. Mean weekly fecal scores for each treatment group are presented in Supplementary Table S5.

### 3.4. Physical Examinations and Adverse Events

Adverse events such as inappetence, vomiting, diarrhea, gingivitis, and alopecia were reported in most cats across all three dietary groups, affecting 13 of 15 cats in both control and Diet B groups, and 14 of 15 cats in the Diet A group. Adverse events were mainly mild with only one serious (fatal) adverse event, as previously described (Table 3). Digestive system issues consisted of mild to severe gingivitis, tartar accumulation, mild abdominal bloat, and vomiting; all cardiovascular system issues were based on observation of pale gums; skin and hair coat problems consisted of individually related self-induced alopecia, and a large skin mass in one individual; and the eye issue recorded in one animal was conjunctivitis. Medication was administered to one cat in the control group (Antisedan, Domitor, Duplocillin, Fresenius Propoven 1%, Ketamine Fresenius 100 mg/mL, Metacam 5 mg/mL, all relating to treatment of a skin mass), on seven occasions in a total of three cats in the Diet A group (Laxapet, Nutrostim gel for inappetence, Chloramex eye ointment for swollen eyelid, Cerenia injection, Laxapet and Nutrostim for treatment of vomiting due to a hairball or foreign object), and to one cat on one occasion (Maxitrol for conjunctivitis) in the Diet B group.

Over the course of the study, there were 32 vomiting instances in the control group and 31 in each of the TE groups. Total number of vomiting events per animal across the study period for the treatment and control groups showed no statistical differences in LMM analysis with group as fixed effect and randomization block as random effect (*p* = 1.000 and 0.849 respectively for Diet A and Diet B groups).

When the total number of adverse events were calculated per animal and the mean numbers of adverse events per group were compared by LS means estimates, there was no significant difference between the treatment and control groups (*p* = 0.888 and 1.000 respectively for the Diet A and Diet B groups).

### 3.5. Complete Blood Count (CBC) and Blood Biochemistry Analyses

**Figure 1:**
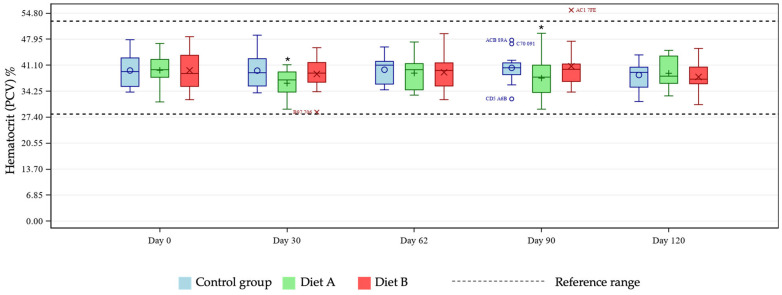
Boxplot showing hematocrit (PCV) % at days 0, 30, 62, 90 and 120, by group. Horizontal lines within boxes represent medians and symbols within the box (o, +, ×) represent means. Outliers are denoted by unique cat code, colored according to treatment group. * denotes significant difference versus control at this timepoint where *p* < 0.10.

**Figure 2:**
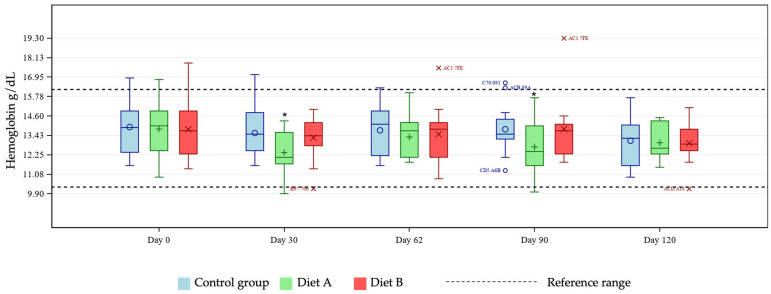
Boxplot showing hemoglobin (g/dL) at days 0, 30, 62, 90 and 120, by group. Horizontal lines within boxes represent medians and symbols within the box (o, +, ×) represent means. Outliers are denoted by unique cat code, colored according to treatment group. * denotes significant difference versus control at this timepoint where *p* < 0.10.

**Figure 3:**
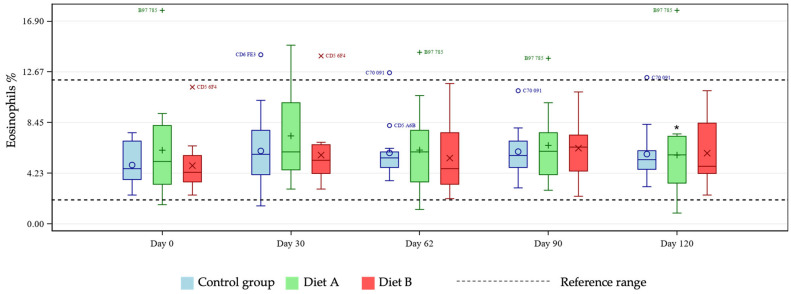
Boxplot showing eosinophils (%) at days 0, 30, 62, 90 and 120, by group. Horizontal lines within boxes represent medians and symbols within the box (o, +, ×) represent means. Outliers are denoted by unique cat code, colored according to treatment group. * denotes significant difference versus control at this timepoint where *p* < 0.10.

Mean CBC marker levels were within reference ranges across all treatment groups (see Supplementary Table S7 for group means at days 0 and 120). A test for fixed effects was significant for the two-way group × day interaction for eosinophils, hematocrit, hemoglobin, lymphocytes (% and concentration), MCH, MCHC, MCV, and platelet count. Pairwise group comparisons by day showed differences between test and control groups at selected timepoints. Hematocrit and hemoglobin were statistically reduced at days 30 and 90 in the Diet A group compared with control (Figure 1 and Figure 2) and eosinophils increased at day 120 in the Diet A group compared with control (Figure 3).

**Figure 4:**
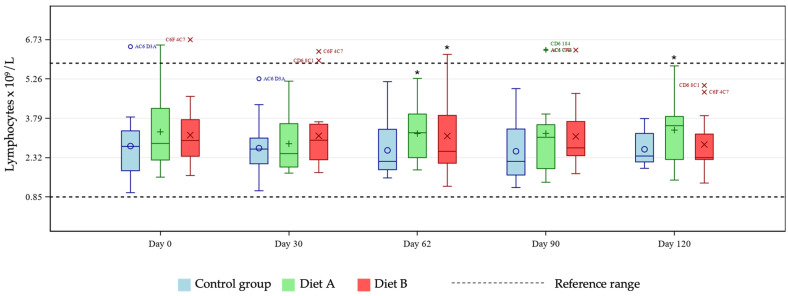
Boxplot showing lymphocyte concentration (×10^9^/L) at days 0, 30, 62, 90 and 120, by group. Horizontal lines within boxes represent medians and symbols within the box (o, +, ×) represent means. Outliers are denoted by unique cat code, colored according to treatment group. * denotes significant difference versus control at this timepoint where *p* < 0.10.

Lymphocyte concentration was significantly increased in both treatment groups at day 62 and in the Diet A group at day 120 compared with control (Figure 4).

**Figure 5:**
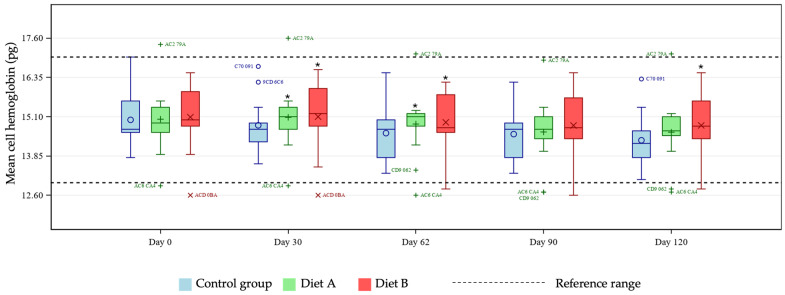
Boxplot showing mean cell hemoglobin (MCH; pg) at days 0, 30, 62, 90, and 120, by group. Horizontal lines within boxes represent medians and symbols within the box (o, +, ×) represent means. Outliers are denoted by unique cat code, colored according to treatment group. * denotes significant difference versus control at this timepoint where *p* < 0.10.

**Figure 6:**
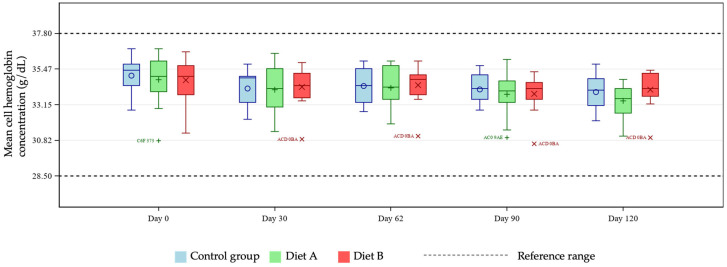
Boxplot showing mean cell hemoglobin concentration (MCHC; g/dL) at days 0, 30, 62, 90 and 120, by group. Horizontal lines within boxes represent medians and symbols within the box (o, +, ×) represent means. Outliers are denoted by unique cat code, colored according to treatment group.

MCH was statistically higher in both diet A and diet B groups compared with control at days 30 and 62, and also in the Diet B group only at day 120 (Figure 5). Group mean MCHC values were significantly higher in the Diet B group at day 30 compared with control (Figure 6).

**Figure 7:**
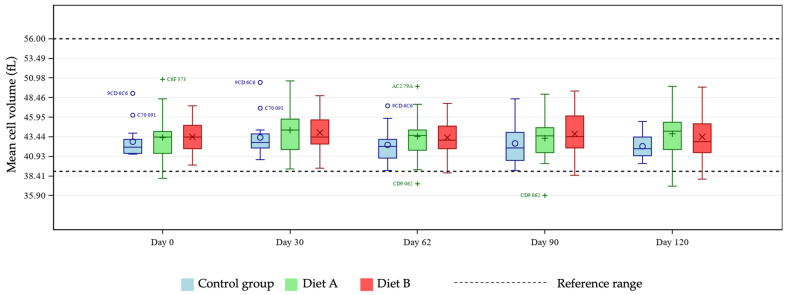
Boxplot showing mean cell volume (MCV; fL) at days 0, 30, 62, 90 and 120, by group. Horizontal lines within boxes represent medians and symbols within the box (o, +, ×) represent means. Outliers are denoted by unique cat code, colored according to treatment group.

MCV was statistically increased at day 62 in the Diet A group compared with control (Figure 7).

**Figure 8:**
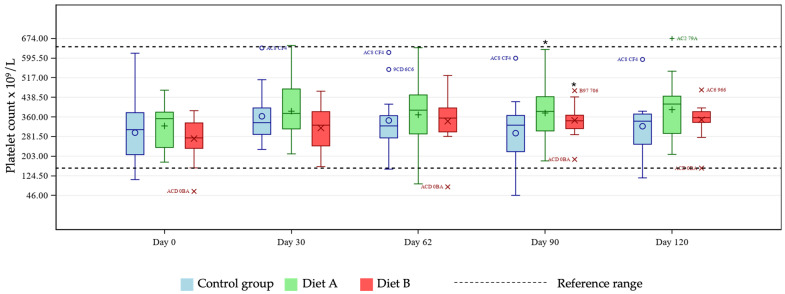
Boxplot showing platelet count ×10^9^/L at days 0, 30, 62, 90, and 120, by group. Horizontal lines within boxes represent medians and symbols within the box (o, +, ×) represent means. Outliers are denoted by unique cat code, colored according to treatment group. * denotes significant difference versus control at this timepoint where *p* < 0.10.

Platelet count was significantly increased in both treatment groups at day 90 compared with control (Figure 8).

Linear plots were generated for individual cats, by group for eosinophils (%), hematocrit, lymphocytes (%) and MCV to further examine the statistical differences in pairwise group comparisons for the Diet A group only (Supplementary Figure S1). None of the differences in these parameters between the Diet A and control groups raised any clinical concerns, with no values outside of acceptable limits of the reference ranges. Linear plots were also generated for lymphocyte concentration, MCH, MCHC and platelet count as these showed some significant differences by day versus control in the Diet B group (Supplementary Figure S2). Inspection of these plots showed that any individual values outside of the reference range were within acceptable limits, with no concerns from a clinical standpoint relating to the treatment.

A test for the main effect of each group was carried out on all parameters showing non-significance in the two-way interaction. A statistical effect of group was observed for basophils, Heinz bodies, monocyte concentration, neutrophil concentration, RBC count, red cell distribution width, and WBC count. Pairwise group comparisons by day were found to be significantly higher for both the Diet A and Diet B groups for basophils, monocyte concentration, neutrophil concentration, and WBC count. Linear plots for individual cats by treatment group revealed similar trends across all three groups for these parameters and no clinically concerning deviations from the reference ranges (Supplementary Figure S3). Statistically lower LS means estimates for Heinz bodies, RBC count, and red cell distribution width were found in the Diet A group only. Linear plots were generated for individual cats by group for these parameters (Supplementary Figure S4). Similar trends were found across all three treatment groups for the three parameters, with RBC count and red cell distribution width values within or close to reference values across all treatment groups. Taken together, any statistical differences in the Diet A group were not deemed to be of clinical significance.

Blood biochemistry parameter means were within reference ranges across all treatment groups, with the exception of globulin and lactate dehydrogenase (see Supplementary Table S8 for means at days 0 and 120). A test for fixed effects showed the group × day interaction to be significant for albumin, amylase, chloride, direct bilirubin, globulin, lactate dehydrogenase, magnesium, potassium, sodium and total serum protein. Pairwise LS means estimates from group comparisons by day were calculated for these parameters. For lactate dehydrogenase, only the Diet A group showed an overall statistical difference (increase) compared with control, with no abnormalities seen in the dataset and all values within acceptable limits of the reference range. The Diet B group showed significant differences for albumin, amylase, chloride, direct bilirubin, globulin, magnesium, potassium, sodium and total serum protein compared with controls. A test for the fixed effect of each group was conducted on all non-significant parameters in the two-way group × day interaction, showing a main effect of group for calcium and glucose (fasting and random). LS means estimates from group comparisons showed only the Diet A group to be statistically lower for calcium and only the Diet B group to be lower for glucose. As for above, closer inspection of the data found no evidence linking these parameters with TE supplementation.

## 4. Discussion

This study examined the safety of TE when applied to dry extruded diet at a level delivering 660 or 1040 ppm total curcuminoids, when fed to healthy adult cats daily for four months. Throughout the study the cats were monitored for BW, BCS, food intake, fecal score, adverse events (including vomiting, diarrhea, clinical signs), CBC including markers of anemia, and blood biochemistry.

The dietary concentrations of TE delivering 660 and 1040 ppm total curcuminoids were selected based on an internal evaluation that considered published feeding studies in cats, official guidance on conducting toxicology studies and toxicologist advice, concluding a presumed safe intake (PSI) of 50 mg total curcuminoids/kg BW/day. Post-extrusion, spray coating application of TE to the kibble was chosen due to the sensitivity of curcuminoids to high temperatures [21,22].

All cats remained relatively healthy throughout the study, with the exception of one fatality in the Diet B group. Based on the clinical summary, the normal CBC and blood chemistry and coagulation parameter results, histopathology of necropsy samples that did not reveal a conclusive etiology or any lesions deemed to be indicative of a toxicity, the most likely cause of death was reported as a cardiac condition or arrhythmia and any link with TE was described as highly unlikely. Every precautionary measure was implemented in the group in which the fatality occurred, as well as in any blood relatives of the deceased cat, to look for evidence of toxicity from the TE. Evaluation of complete lab work for all subjects up to and including Day 90 of the study did not reveal any significant abnormalities that would suggest evidence of a bleeding disorder or other toxic change. All subjects maintained normal hematocrit, had no elevations in their reticulocyte count, and no evidence of hepatotoxicity within any of the test groups. Additional blood analyses (coagulation parameters: PT, PTT, Fibrinogen), were performed just after Day 120, on all cats within the same group of the deceased subject, as well as any cats genetically related to the cat within the facility. No evidence of this issue was detected, making the TE an unlikely contributor to this unfortunate situation.

There were no statistically important effects of the test diets on bodyweight, BCS, fecal score, food intake or adverse events, which included vomiting episodes. These findings show that addition of TE to the daily diet is well tolerated and did not compromise food intake.

The unique hemoglobin structure in cats that predisposes them to reduced oxygen affinity represents an area of potential concern, considering reports in other species linking curcumin with anemia [16,17]. Several tests exist for diagnosing anemia in cats, with no single optimal test available [23,24,25,26]. For these reasons, this study tested for any effects of the two dietary levels of TE used on several markers of anemia, namely hematocrit, hemoglobin, MCH, MCHC, MCV, and Heinz bodies. At days 30 and 90, pairwise group comparisons showed a statistically significant reduction in hematocrit and hemoglobin means in the Diet A group. Further investigation of individual cat data showed all values to be within reference ranges [19,20], demonstrating no clinically meaningful issues relating to TE supplementation in the population studied. Any individual hemoglobin values outside of the reference range in this study were at the upper end of the range in the treatment groups, giving no cause for concern relating to the addition of TE. There is no reference range for Heinz bodies in cats as they are expected to be present in very low numbers in healthy individuals. An increase in Heinz body formation is indicative of oxidative damage and is therefore an important measure of toxicity in cats. In the current study there was a significantly lower mean % Heinz body formation in the Diet A group but not in the Diet B group compared with control. These observations confirm that addition of TE to a feline diet is not associated with increased Heinz bodies compared to the unsupplemented diet. The lack of progressive decline in MCH and absence of MCH levels below the reference range suggests no clinically relevant issues. In the Diet B group, one individual started and finished the study close to the low end of the MCH reference range, remaining relatively stable across the course of the study. Despite a significantly increased group mean of MCHC at day 30 in the Diet B group compared with control, all cats remained within the reference range, supporting the safety of the supplementation, as this did not represent a clinically meaningful change. Similarly, individual MCV values were within the reference range for all cats except for one individual in the Diet A group. That subject showed levels slightly below the lower limit of the reference range, which likely drove the statistical significance of the group mean comparisons at day 62 in the Diet A group compared with control. Also likely contributing to this was the broader variability in MCV values within the Diet A group compared to a tighter distribution seen in the control cats. All data were evaluated by a veterinary internal medicine specialist and found to be without clinical concern. Overall, the study has shown no clinically relevant effects of TE supplementation in healthy cats in terms of markers of anemia.

Several hematological parameters (eosinophils, hematocrit, hemoglobin, lymphocytes (%) and MCV) showed statistically significant differences in the Diet A group versus control, but not in the Diet B group. This suggests no biologically meaningful influence of the TE on the outcomes, and the fact the values remained within reference ranges provides further confirmation of safety. This lack of dose effect was apparent throughout the study across several measured parameters and suggests a degree of random variation which could not be attributed to dietary levels of TE. Cat hemoglobin has 8–10 readily oxidizable sulfhydryl groups, four times more than found in dogs and humans [27,28]—this renders feline erythrocytes particularly sensitive to oxidative damage from exposure to certain phenolic compounds. In light of this, hematological parameters are considered the most relevant endpoints for cats in terms of demonstrating a lack of species-specific effects relating to ingestion of TE. A small selection of TE toxicity studies have been conducted in rats. Feeding highly available curcumin to rats for 28 days at a dose level of up to 3500 mg/kg BW/day resulted in no mortality or systemic toxicity, with evidence of some modest, non-adverse variations in clinical chemistry parameters [29]. All other parameters, including BW, food intake, hematology, organ weights (except for a small, non-adverse liver-weight increase in high-dose females), and gross pathology, were comparable to controls. In a longer-term study of highly available TE in rats, there were no adverse effects on BW, food intakes, clinical pathology parameters, macroscopic necropsy or histopathological tissue examination at the highest dose of 3000 mg/kg BW/day over 90 days [30]. A reproductive toxicity study showed lack of adverse toxicological effects of curcumin fed to two successive generations of rats at up to 1043 mg/kg BW/day, with no gross or microscopic organ changes observed [14]. Blood biochemistry parameters that were significantly different in the Diet B group compared with control were inspected more closely. When outliers, individual values relative to the reference ranges, and evidence for any concerning progression were considered, no issues associated with TE were found from a clinical standpoint.

Liver function markers ALT, ALP, AST, bilirubin, GGT and albumin were examined in the current study to evaluate any toxic effects of the TE. Elevated ALT, ALP, AST and GGT levels are indicative of liver damage and the current study demonstrated no significant effect of treatment group on these parameters, with all values falling within their relative reference ranges. Total bilirubin levels showed no significant association with treatment at any timepoint. The significantly higher mean levels of direct bilirubin at day 62 in both test diet groups compared with control, and significantly lower levels at day 120 in the Diet A group only were investigated in terms of clinical relevance since increased levels can indicate abnormal liver function. It was found that cats across all groups showed similar trends in changes to direct bilirubin levels, with an increase in mean levels at day 30 followed by a gradual decline from days 62 to 120. No individual showed progressive increases in bilirubin across the course of the study which indicates absence of accumulation. Reduced serum albumin is another indicator of impaired liver function. At day 90, mean serum albumin levels were significantly lower in the Diet B group versus controls. This was driven by two individuals in the group that displayed levels slightly below the minimum reference level. However, serum albumin levels in these individuals recovered at day 120 to within reference range to give no overall group difference versus control at this timepoint. This suggests that the reduced albumin levels seen at a single timepoint in two subjects were not progressive in nature and it was concluded that the effect was not clinically significant. Overall, the results from this set of measures provide evidence that the two levels of TE ingested daily over the course of four months did not show any clinically relevant adverse effects on liver function.

To the authors’ knowledge, the current study is the first to test TE in cats at the levels used, for the sole purpose of safety evaluation. Compared to previously reported feeding studies of TE in cats, the current study tested higher doses of TE, equating to approximately double (156 mg/1000 kcal) and triple (246 mg/1000 kcal) the 83 mg/1000 kcal level fed to cats as part of a combination of functional ingredients designed to alleviate symptoms of osteoarthritis [7]. Based on mean daily food intakes for each group, mean daily total curcuminoid intakes in the current study were 35.7 mg in the Diet A group and 54.2 mg in the Diet B group, equating to 8.5 and 12.9 mg/kg BW/day. Again, these are higher levels than the daily intakes of approximately 28.5 mg (4.75 mg/kg BW/day) in a study in which cats received direct oral dosing for 10 weeks [8].

One of the key strengths of this study is the randomized, controlled, blinded design which ensures minimal introduction of bias and increases confidence in the data. The substantial set of measures is another strength, enabling a thorough and relevant safety evaluation of TE in the target species. The rigorous statistical analyses, conducted according to official guidelines for analysis of toxicology studies, have ensured a thorough evaluation of the outcomes, enabling confidence regarding the safety of TE. Whilst the statistical approach is a strength of the study, the increased sensitivity of setting a significance level of 0.10 can increase Type I errors and highlight apparent differences that, when analyzed further, may have no biological or physiological relevance. The delivery of TE as part of a dry kibble diet in this study rather than direct oral dosing allowed assessment in a format representative of the intended application, further strengthening the relevance of the findings. Application to a kibble diet also demonstrated that, with careful formula adaptation involving the use of a highly palatable fat, addition of TE in the intended application format did not compromise palatability. TE is known to have a pungent smell and bitter taste; the standardized palatant system used in all three diets ensured uniform acceptance, as evidenced by the lack of dietary effect on daily food intakes and BW. Delivery of functional ingredients to pets within the diet, rather than as an orally dosed supplement, encourage compliance and therefore promote the likelihood of an associated benefit. It should be acknowledged that the study was conducted in colony-housed cats; direct extrapolation of the findings to pet cats is expected despite some subtle differences in living conditions relative to client-owned pet cats. Similarly, application of the findings is currently limited to healthy adult cats and is not transferable to cats in disease states or to other life stages, i.e., kittens or senior cats. The intense pigment associated with the TE gave a yellow coloration to the two TE diets, potentially preventing full blinding of the study. However, the personnel involved in feeding the cats did not take part in any of the key assessments, therefore we believe any compromise to blinding was minimal.

Increasing interest and awareness of pet nutrition and wellbeing has driven a demand by consumers for good quality, natural, functional pet food products that go beyond providing essential nutrition [31,32]. The safety of companion animal foods, especially those containing novel ingredients, has also become a key focus of consumers [33]. TE in combination with other functional ingredients has shown some promise in limited numbers of efficacy studies in cats, yet there are few data relating to the safety of this ingredient in this species. The current study contributes towards a much-needed knowledge base around the safety of TE in cats, opening up opportunities for inclusion of functionally beneficial levels of this ingredient in cat food.

## 5. Conclusions

This study confirms that inclusion of TE up to a level of 1040 ppm total curcuminoids in complete and balanced cat food is well tolerated when fed in a study of 45 healthy adult domesticated cats daily for a period of 120 days. This conclusion was based on the lack of significant effects of TE on several key parameters including clinical signs, markers of anemia, and markers of liver damage and function. These findings support safe application of the tested level of TE in adult feline nutrition.

## Figures and Tables

**Table 1:** Summary of baseline characteristics of animals, by dietary group.

	Statistic	Control Group (*N* = 15)	Diet A (*N* = 15)	Diet B (*N* = 15)
**Day 0 age (years)**	Mean	4.9	5.0	4.9
	SD	1.44	1.46	1.60
	Median	5.0	5.0	6.0
	Range	2.0–7.0	2.0–8.0	2.0–6.0
**Day 0 bodyweight (kg)**	Mean	4.17	4.21	4.22
	SD	0.67	0.66	0.67
	Median	4.10	4.20	4.15
	Range	3.0–5.3	3.1–5.4	3.2–5.4
**Sex/reproductive status**				
**Female**	n/*N* (%)	7/15 (46.7)	6/15 (40)	6/15 (40)
**Intact**	n/*N*f (%)	1/7 (14.3)	1/6 (16.7)	1/6 (16.7)
**Neutered**	n/*N*f (%)	6/7 (85.7)	5/6 (83.3)	5/6 (83.3)
**Male**	n/*N* (%)	8/15 (53.3)	9/15 (60.0)	9/15 (60.0)
**Intact**	n/*N*m (%)	0/8 (0)	1/9 (11.1)	1/9 (11.1)
**Neutered**	n/*N*m (%)	8/8 (100)	8/9 (88.9)	8/9 (88.9)

*N*f refers to number of female cats; *N*m refers to number of male cats.

**Table 2:** Standard analyses and ingredients of study diets.

	Control Diet	Diet A	Diet B
Protein (%)	38.0	39.0	37.9
Fat (%)	17.9	18.5	17.8
Crude fiber (%)	<2.0	<2.0	<2.0
Moisture (%)	5.7	5.7	5.7
Total curcuminoids (ppm)	-	660	1040
Energy (kcal/kg)	4231	4231	4228

Dehydrated poultry proteins, maize flour, wheat flour, animal fats, wheat gluten, rice, egg powder, hydrolyzed animal proteins, minerals, beet pulp, yeast products, soya oil. Nutritional additives: vitamin A: 9900 IU, vitamin D3: 500 IU, iron: 15 mg, iodine: 1.5 mg, copper: 5 mg, manganese: 19 mg, zinc: 123 mg, selenium: 0.04 mg. Technological additives: sodium bisulfate: 3 g—antioxidants.

**Table 3:** Categories of physical examination findings recorded in cats during the 4-month study, by dietary group.

Abnormal Clinical Observation	Number of Cats Affected in Each Dietary Group		
	Control	Diet A	Diet B
Digestive system	34	33	37
Cardiovascular system	3	6	5
Skin/hair	4	4	1
Eyes	0	1	0

## Data Availability

The data associated with the article will be shared upon reasonable request to the corresponding author. The data are not publicly available because a regulatory file is in progress.

## Supplementary Materials

The following supporting information can be downloaded at: https://www.mdpi.com/article/10.3390/ani16091355/s1, Table S1: Details of individual housing used in the study; Table S2: Analysis of total curcuminoids (curcumin, desmethoxycurcumin and bisdemethoxycurcumin) in finished study diets; Table S3: System used in the current study for assessment of body condition score (BCS) in cats; Table S4: Mean bodyweights (kg) by study week for control, diet A and diet B groups; Table S5: Mean food intake (g/day) by study week for control, diet A and diet B groups; Table S6: Mean fecal scores by study week for control, diet A and diet B groups; Table S7: Group mean complete blood count parameters at start (day 0) and end of study (day 120) for control and treatment cats. Table S8: Group mean blood biochemistry parameters at start (day 0) and end of study (day 120) for control and treatment cats. Figure S1: Linear plots of complete blood count parameters for individual cats by treatment group showing (a) eosinophils (%); (b) hematocrit (PCV) (%); (c) hemoglobin (g/dL); (d) lymphocytes (%); (e) mean cell volume (MCV) (%), at days 0, 30, 62, 90 and 120; Figure S2: Linear plots of complete blood count parameters for individual cats by treatment group showing (a) lymphocytes (×10^9^/L) (b) MCH (mean cell hemoglobin) (pg); (c) MCHC (mean cell hemoglobin concentration) (g/dL); (d) platelet count, at days 0, 30, 62, 90 and 120. Figure S3: Linear plots of complete blood count parameters for individual cats by treatment group showing (a) basophils (×10^9^/L); (b) monocytes (×10^9^/L); (c) neutrophils (×10^9^/L); (d) white blood cell (WBC) count (×10^9^/L) at days 0, 30, 62, 90 and 120. Figure S4: Linear plots of complete blood count parameters for individual cats by treatment group showing (a) Heinz bodies (%); (b) RBC (red blood cell) count (%); (c) RCDW (red cell distribution width) (%) at days 0, 30, 62, 90 and 120.

## Abbreviations

The following abbreviations are used in this manuscript:

AAALAC	Association for Assessment and Accreditation of Laboratory Animal Care
AAFCO	Association of American Feed Control Officials
ALP	Alkaline phosphatase
ALT	Alkaline transferase
AST	Aspartate aminotransferase
BCS	Body condition score
BW	Bodyweight
CBC	Complete blood count
FDA	Food and Drug Administration
GGT	Gamma-glutamyl transferase
GLM	Generalized linear model
IACUC	Institutional Animal Care and Use Committee
LMM	Linear mixed model
LS	Least squares
MCH	Mean cell hemoglobin
MCHC	Mean cell hemoglobin concentration
MCV	Mean cell volume
PSI	Presumed safe intake
PT	Prothrombin time
PTT	Partial thromboplastin time
RBC	Red blood cell
TE	Turmeric extract
TSP	Total serum protein
VIHC	Veterinary International Cooperation on Harmonization
WBC	White blood cell
