# Supplementary material for: A Tolerance Study of Turmeric Extract in Healthy Adult Cats
Source: Animals (Basel). 2026 Apr 28;16(9):1355. doi: 10.3390/ani16091355 (PMC13162889; doi:10.3390/ani16091355)
Supplement: Supplementary file 1 [file animals-16-01355-s001.zip › animals-4228375-supplementary.pdf]

Supplementary Information

Table S1. Details of individual housing used in the study.

	Enclosed sleeping area				Open exercise area			
**Unit number**	**Surface area (m^2)**	**Width (m)**	**Length (m)**	**Height (m)**	**Surface area (m^2)**	**Width (m)**	**Length (m)**	**Height (m)**
1	2.40	1.50	1.60	2.0	4.50	1.50	3.0	2.0
2 [1]	1.33 – 2.31	0.94 – 1.52	1.41 – 1.52	2.0	2.51 – 4.62	0.94 – 1.52	2.66 – 3.04	2.0

[1] cages in unit 2 vary in size therefore dimensions are given as ranges. Individual housing consisted of steel cages containing concrete floors, an outside sleeping bench and an enclosed sleeping area containing benches. Bedding was not provided in the housing. The environment was enriched via raised sleeping and resting areas, scratch pads, a tennis ball fixed to a string and at least one toy which was replaced weekly. Cats could see and hear one another within their units but no physical cross-contact was possible.

Table S2. Analysis of total curcuminoids (curcumin, desmethoxycurcumin and bisdemethoxycurcumin) in finished study diets.

Diet	Mean total curcuminoids [1] (g/100g)	SD	CV%	Uncertainty (g/100g)	Measured dose (ppm)	Theoretical target dose (ppm)	Deviation (% total ppm)
**Control**	< LoQ	NC	NC	NC	< LoQ	NC	NC
**Diet A**	0.0660	0.0008	1.16	0.0008	660	600	9.94
**Diet B**	0.1040	0.0017	1.67	0.0083	1040	1200	-13.33

[1] Means calculated from triplicate analysis results. NC: not calculated. LoQ: limit of quantification

Table S3. System used in the current study for assessment of body condition score (BCS) in cats.

Score	Description	Body condition
**1**	Ribs visible on shorthaired cats. No palpable fat. Severe abdominal tuck. Lumbar vertebrae and wings of ilia easily palpated.	
**2**	Ribs easily visible on shorthaired cats. Lumbar vertebrae obvious. Pronounced abdominal tuck. No palpable fat.	Under ideal
**3**	Ribs easily palpable with minimal fat covering. Lumbar vertebrae obvious. Obvious waist behind ribs. Minimal abdominal fat.	
**4**	Ribs palpable with minimal fat covering. Noticeable waist behind ribs. Slight abdominal tuck. Abdominal fat pad absent.	
**5**	Well-proportioned. Observe waist behind ribs. Ribs palpable with slight fat covering. Abdominal fat pad minimal.	Ideal
**6**	Ribs palpable with slight excess fat covering. Waist and abdominal fat pad distinguishable but not obvious. Abdominal tuck absent.	
**7**	Ribs not easily palpated with moderate fat covering. Waist poorly discernible. Obvious rounding of abdomen. Moderate abdominal fat pad.	
**8**	Ribs not palpable with excess fat covering. Waist absent. Obvious rounding of abdomen with prominent abdominal fat pad. Fat deposits present over lumbar area.	Over ideal
**9**	Ribs not palpable under heavy fat cover. Heavy fat deposits over lumbar area, face and limbs. Distention of abdomen with no waist. Extensive abdominal fat deposits.	

Table S4. Mean bodyweights (kg) by study week for control, diet A and diet B groups.

**Group**	**Statistic**	**0**	**1**	**2**	**3**	**4**	**5**	**6**	**7**	**8**	**9**	**10**	**11**	**12**	**13**	**14**	**15**	**16**	**17**
Control	n	15	15	15	15	15	14	13	13	13	13	13	13	13	13	12	12	12	12
	Mean	4.17	4.18	4.2	4.21	4.21	4.26	4.37	4.38	4.36	4.35	4.40	4.41	4.42	4.38	4.43	4.47	4.42	4.39
Diet A	n	15	15	15	15	15	15	15	15	15	15	15	14	14	14	14	14	14	14
	Mean	4.21	4.23	4.24	4.28	4.27	4.27	4.28	4.28	4.34*	4.34*	4.35*	4.33*	4.32*	4.30*	4.30*	4.29	4.30*	4.24
Diet B	n	15	15	15	15	15	15	14	14	14	14	14	14	14	13	13	13	13	13
	Mean	4.22	4.18	4.26	4.24	4.29	4.27	4.35	4.38*	4.36*	4.35	4.38	4.38	4.40	4.29	4.29	4.31	4.32	4.26

* denotes significant difference ($p < 0.10$) compared with control value in same week

Table S5. Mean food intake (g/day) by study week for control, diet A and diet B groups.

Group	Statistic	1	2	3	4	5	6	7	8	9	10	11	12	13	14	15	16	17
Control	n	15	15	15	15	15	14	13	13	13	13	13	13	13	13	12	12	12
	Mean	46.5	47.1	47.8	48.5	41.6	50.0	52.0	52.3	50.5	53.7	52.9	50.2	49.7	52.6	53.7	53.2	49.4
Diet A	n	15	15	15	15	15	15	15	15	15	15	15	14	14	14	14	14	14
	Mean	51.1	51.7	52.8	50.8	48.6	53.8	54.4	54.4	51.4	53.1	47.9	49.7	49.3	51.1	53.3	53.3	50.1
Diet B	n	15	15	15	15	15	15	14	14	14	14	14	14	14	13	13	13	13
	Mean	49.5*	48.3	48.9	48.2	45.0	51.2	52.2	52.2	48.9	52.1	51.0	49.3	48.5	50.6	50.9	50.8	46.6

* denotes significant difference ($p < 0.10$) compared with control value in same week

Table S6. Mean fecal scores by study week for control, diet A and diet B groups.

Group	Statistic	1	2	3	4	5	6	7	8	9	10	11	12	13	14	15	16	17
Control	n	15	15	15	15	15	14	13	13	13	13	13	13	13	13	12	12	12
	Mean	2.31	2.33	2.33	2.38	2.36	2.54	2.54	2.53	2.50	2.41	2.48	2.42	2.56	2.50	2.57	2.53	2.54
Diet A	n	15	15	15	15	15	15	15	15	15	15	14	14	14	14	14	14	14
	Mean	2.47	2.51*	2.54*	2.44	2.42	2.65	2.64	2.59	2.52	2.51	2.58	2.58	2.67	2.62	2.62	2.71*	2.68
Diet B	n	15	15	15	15	15	15	14	14	14	14	14	14	14	13	13	13	13
	Mean	2.34	2.39	2.38	2.43	2.25*	2.52	2.56	2.57	2.55	2.49	2.47	2.57	2.57	2.58	2.55	2.64	2.60

* denotes significant difference ($p < 0.10$) compared with control value in same week

Table S7. Group mean complete blood count parameters at start (day 0) and end of study (day 120) for control and treatment cats.

Variable (units)	Reference range	Day 0			Day 120		
		Control	Diet A	Diet B	Control	Diet A	Diet B
Basophils (%)	0–3	0.29 ± 0.168	0.19 ± 0.122	0.11 ± 0.119	0.11 ± 0.124	0.18 ± 0.142	0.18 ± 0.06
Basophils number (x 10^9/L)	0–0.1	0.022 ± 0.014	0.019 ± 0.016	0.012 ± 0.012	0.009 ± 0.01	0.016 ± 0.015	0.014 ± 0.005
Eosinophils (%)	2.0–12	4.91 ± 1.71	6.14 ± 3.988	4.85 ± 2.194	5.82 ± 2.415	5.74 ± 4.110	5.89 ± 2.856
Eosinophils number (x 10^9/L)	0.09–2.18	0.376 ± 0.158	0.542 ± 0.323	0.412 ± 0.137	0.400 ± 0.191	0.514 ± 0.365	0.488 ± 0.264
Hematocrit (%)	28.2–52.7	39.68 ± 4.265	39.75 ± 4.639	39.72 ± 4.892	38.50 ± 3.691	38.95 ± 4.001	37.99 ± 3.918
Hemoglobin (g/dL)	10.3–16.2	13.93 ± 1.722	13.81 ± 1.617	13.79 ± 1.691	13.10 ± 1.533	12.98 ± 1.078	12.95 ± 1.253
Heinz bodies (%)	-	0 ± 0	0 ± 0	0 ± 0	0.083 ± 0.123	0.071 ± 0.153	0.038 ± 0.094
Immature reticulocyte fraction (%)	-	37.97 ± 19.468	40.85 ± 26.853	33.75 ± 16.48	38.75 ± 18.759	39.52 ± 21.677	31.57 ± 15.569
Lymphocytes (%)	20–55	34.69 ± 10.336	34.72 ± 9.805	36.07 ± 12.355	39.22 ± 8.887	36.62 ± 8.581	33.45 ± 10.67
Lymphocytes x 10^9/L	0.85–5.85	2.752 ± 1.308	3.282 ± 1.472	3.163 ± 1.257	2.632 ± 0.643	3.349 ± 1.163	2.811 ± 1.14
MCH (pg)	13–17	15.00 ± 0.791	15.02 ± 0.982	15.09 ± 1.008	14.35 ± 0.874	14.61 ± 1.054	14.82 ± 1.094
MCHC (g/dL)	28.5–37.8	35.05 ± 1.07	34.78 ± 1.527	34.75 ± 1.325	33.98 ± 1.146	33.40 ± 1.124	34.13 ± 1.228
MCV (fL)	39–56	42.80 ± 2.169	43.33 ± 3.395	43.42 ± 2.169	42.22 ± 1.556	43.81 ± 3.378	43.42 ± 2.968
Monocytes (%)	1.0–4.0	3.55 ± 1.148	3.29 ± 1.092	3.31 ± 0.911	2.96 ± 1.200	3.22 ± 1.096	2.97 ± 0.753
Monocytes (x 10^9/L)	0.04–0.53	0.28 ± 0.133	0.309 ± 0.152	0.295 ± 0.114	0.210 ± 0.111	0.304 ± 0.168	0.254 ± 0.108
Neutrophils (%)	35–75	56.57 ± 9.380	55.65 ± 9.955	55.65 ± 12.585	51.90 ± 8.026	54.24 ± 9.780	57.52 ± 9.627
Neutrophils (x 10^9/L)	2.62–15.2	4.425 ± 1.514	5.163 ± 1.773	5.167 ± 2.63	3.643 ± 1.278	4.924 ± 1.550	4.817 ± 1.419
Platelet count (x 10^9/L)	155–641	296.9 ± 131.51	323.9 ± 93.99	273.4 ± 83.87	322.8 ± 116.19	389.0 ± 130.18	348.7 ± 72.67
RBC count (x 10^{12}/L)	7.12–11.46	9.287 ± 1.062	9.213 ± 1.225	9.193 ± 1.392	9.115 ± 0.752	8.922 ± 1.006	8.811 ± 1.262
Red cell distribution width (%)	16–24	18.85 ± 1.195	18.81 ± 1.168	18.43 ± 1.565	17.93 ± 1.000	17.50 ± 1.177	17.67 ± 1.303
Reticulocyte (x 10^9/L)	9–61	15.63 ± 10.183	13.79 ± 7.812	15.99 ± 6.904	16.36 ± 12.644	18.48 ± 12.709	21.41 ± 11.828
Reticulocytes (%)	0.1–0.7	0.17 ± 0.113	0.149 ± 0.082	0.174 ± 0.076	0.177 ± 0.132	0.205 ± 0.138	0.239 ± 0.115
Reticulocytes hemoglobin (pg)	13.2–20.8	16.85 ± 1.143	16.39 ± 1.924	16.55 ± 0.928	15.79 ± 1.07	15.78 ± 1.218	16.05 ± 1.007
WBC count (x 10^9/L)	3.9–19	7.855 ± 2.499	9.315 ± 2.729	9.049 ± 2.902	6.893 ± 1.638	9.106 ± 2.447	8.383 ± 1.853

Data are presented as mean ± standard deviation.

Table S8. Group mean blood biochemistry parameters at start (day 0) and end of study (day 120) for control and treatment cats.

Variable (units)	Reference range	Day 0			Day 120		
		Control	Diet A	Diet B	Control	Diet A	Diet B
ALT (U/L)	27–158	63.435 ± 17.705	63.981 ± 24.830	56.85 ± 15.087	59.799 ± 17.681	56.786 ± 17.712	51.012 ±13.792
Albumin (g/L)	26–39	31.354 ± 2.458	29.727 ± 1.851	30.526 ± 2.871	30.559 ± 1.91	28.917 ± 2.074	29.188 ± 2.598
ALP (U/L)	12–59	26.668 ± 11.877	26.047 ± 10.000	28.287 ± 9.625	25.97 ± 10.964	29.39 ± 11.360	28.563 ± 12.069
Amylase (U/L)	623–2239	986.294 ± 132.592	1115.190 ± 201.792	1041.731 ± 260.971	921.126 ± 84.24	1157.861 ± 204.619	1082.21 ± 240.091
AST (U/L)	16–67	31.614 ± 7.861	26.58 ± 8.404	28.429 ± 16.264	27.977 ± 5.441	26.138 ± 5.814	26.775 ± 12.978
Calcium (mmol/L)	2–2.8	2.361 ± 0.105	2.359 ± 0.093	2.338 ± 0.062	2.373 ± 0.126	2.265 ± 0.255	2.331 ± 0.071
Chloride (mmol/L)	114–126	117.907 ± 1.807	119.419 ± 1.856	117.092 ± 1.491	118.082 ± 2.169	118.353 ± 1.673	117.43 ± 1.565
Cholesterol (mmol/L)	2.4–7.9	4.108 ± 1.522	4.095 ± 1.133	4.027 ± 0.83	4.565 ± 1.437	4.146 ± 0.965	4.555 ± 1.128
Creatine kinase (U/L)	64–440	453.937 ± 539.045	238.158 ± 123.455	354.25 ± 498.975	274.154 ± 183.945	236.636 ± 129.889	371.075 ± 353.762
Creatinine (μmol/L)	80–203	112.617 ± 19.409	107.498 ± 20.965	117.829 ± 24.35	118.25 ± 14.576	111.886 ± 22.079	119.569 ± 21.649
Direct bilirubin (μmol/L)	-	0.172 ± 0.253	0.093 ± 0.118	0.105 ± 0.121	0.445 ± 0.237	0.326 ± 0.177	0.518 ± 0.169
GGT (U/L)	0–6	0 ± 0	0 ± 0	0 ± 0	0 ± 0	0.008 ± 0.029	0.052 ± 0.132
Globulin (g/L)	25–38	41.639 ± 4.632	43.263 ± 6.536	44.760 ± 5.62	39.459 ± 4.474	42.239 ± 5.78	43.976 ± 5.082
Glucose (fasting or random) (mmol/L)	4–9.7	5.181 ± 1.259	4.577 ± 0.556	4.903 ± 1.237	4.153 ± 0.306	4.109 ± 0.675	4.065 ± 0.396
Lactate dehydrogenase (U/L)	161–1051	144.939 ± 69.736	91.269 ± 41.557	91.205 ± 37.374	156.39 ± 67.538	162.502 ± 65.017	131.785 ± 43.665
Magnesium (mmol/L)	0.74–1	0.848 ± 0.044	0.843 ± 0.041	0.844 ± 0.041	0.866 ± 0.038	0.823 ± 0.044	0.842 ± 0.061
Phosphorus (inorganic) (mmol/L)	0.93–2	1.387 ± 0.182	1.529 ± 0.243	1.405 ± 0.154	1.443 ± 0.153	1.419 ± 0.222	1.405 ± 0.155
Potassium (mmol/L)	3.7–5.2	4.257 ± 0.281	4.331 ± 0.300	4.201 ± 0.471	4.445 ± 0.283	4.745 ± 0.701	4.427 ± 0.240
Sodium (mmol/L)	147–157	150.485 ± 1.575	152.131 ± 1.514	150.163 ± 1.511	151.488 ± 1.247	151.454 ± 1.964	150.697 ± 1.128
Total bilirubin (μmol/L)	-	2.813 ± 0.258	2.943 ± 0.424	2.904 ± 0.273	2.640 ± 0.284	2.557 ± 0.312	2.682 ± 0.262
TSP (g/L)	63–88	72.993 ± 4.154	72.991 ± 5.832	75.286 ± 4.665	70.018 ± 4.057	71.156 ± 4.974	73.165 ± 5.237
Urea (mmol/L)	5.7–13.2	7.971 ± 1.263	7.560 ± 0.835	7.969 ± 1.402	8.203 ± 0.847	7.504 ± 1.106	7.432 ± 1.251

Data are presented as mean ± standard deviation.

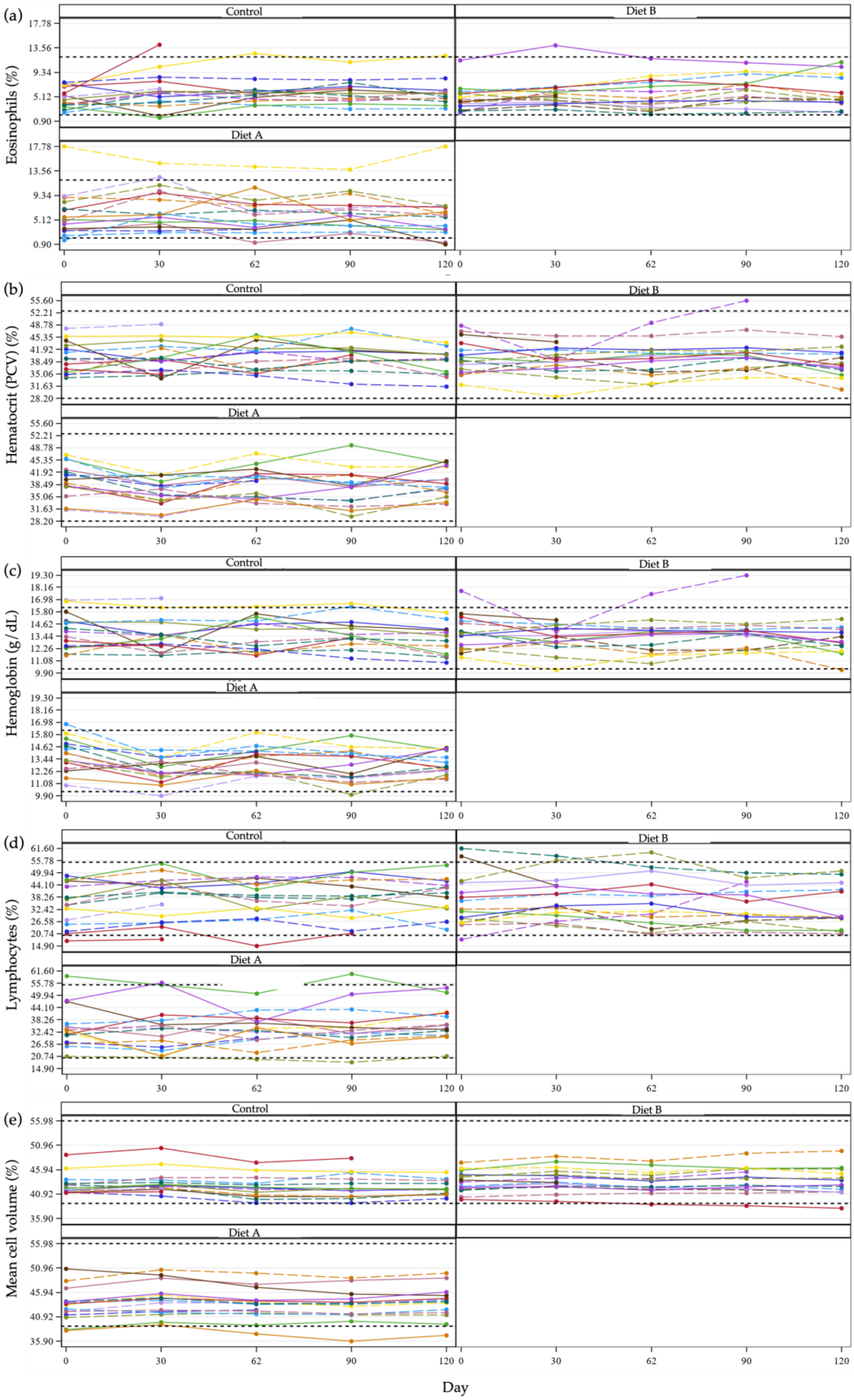

Figure S1. Linear plots of complete blood count parameters for individual cats by treatment group showing (a) eosinophils (%); (b) hematocrit (PCV) (%); (c) hemoglobin (g/dL); (d) lymphocytes (%); (e) mean cell volume (MCV) (%), at days 0, 30, 62, 90 and 120. These parameters showed significant differences in pairwise

group mean comparisons by day between Diet A and control groups only. - - - - - - - - - denotes reference ranges.

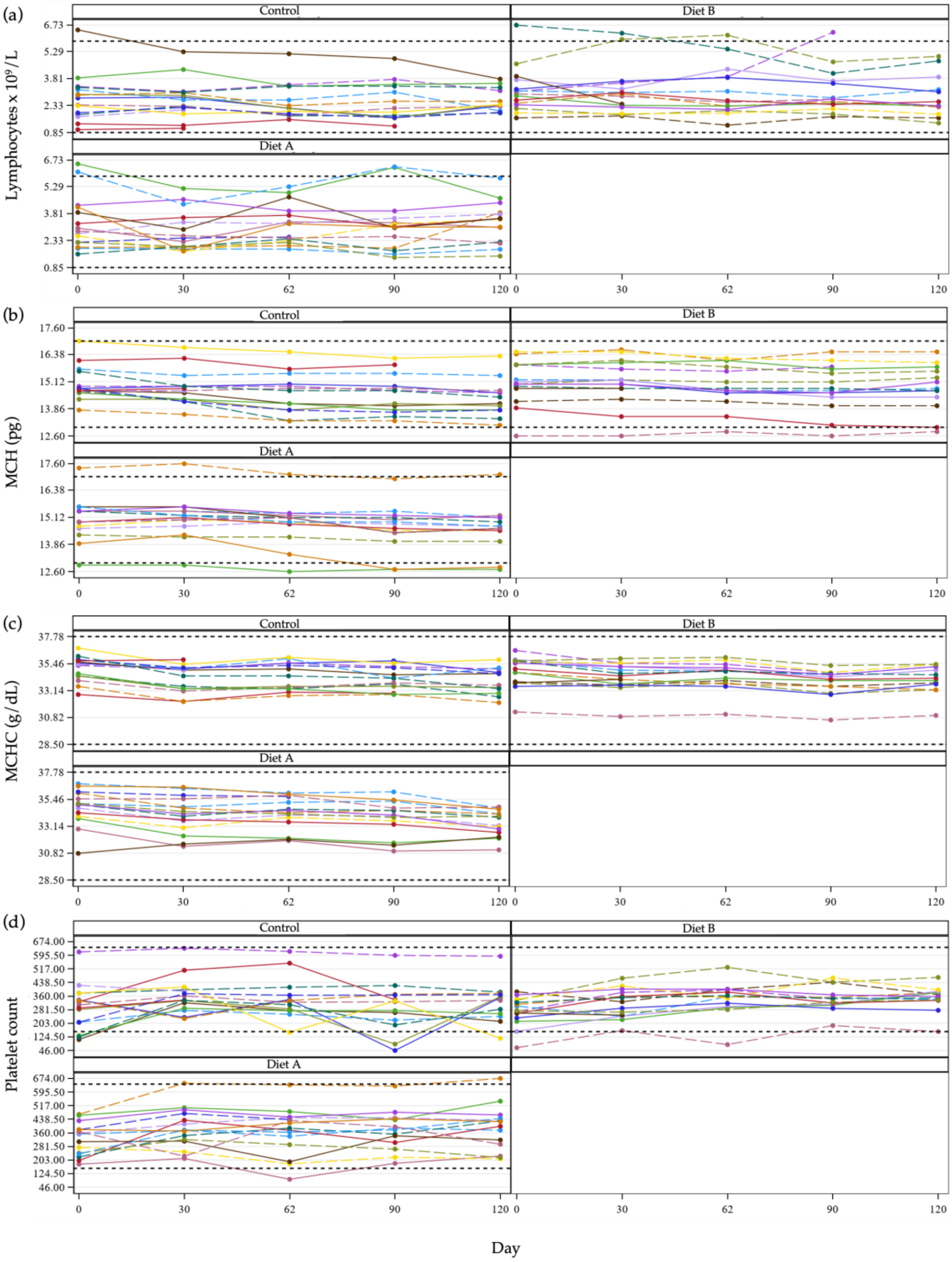

Figure S2. Linear plots of complete blood count parameters for individual cats by treatment group showing (a) lymphocytes (x 10^9/L) (b) MCH (mean cell hemoglobin) (pg); (c) MCHC (mean cell hemoglobin concentration) (g/dL); (d) platelet count, at days 0, 30, 62, 90 and 120. These parameters showed significant differences in pairwise group mean comparisons by day between Diet B and control groups. - - - - - - - - - denotes reference ranges.

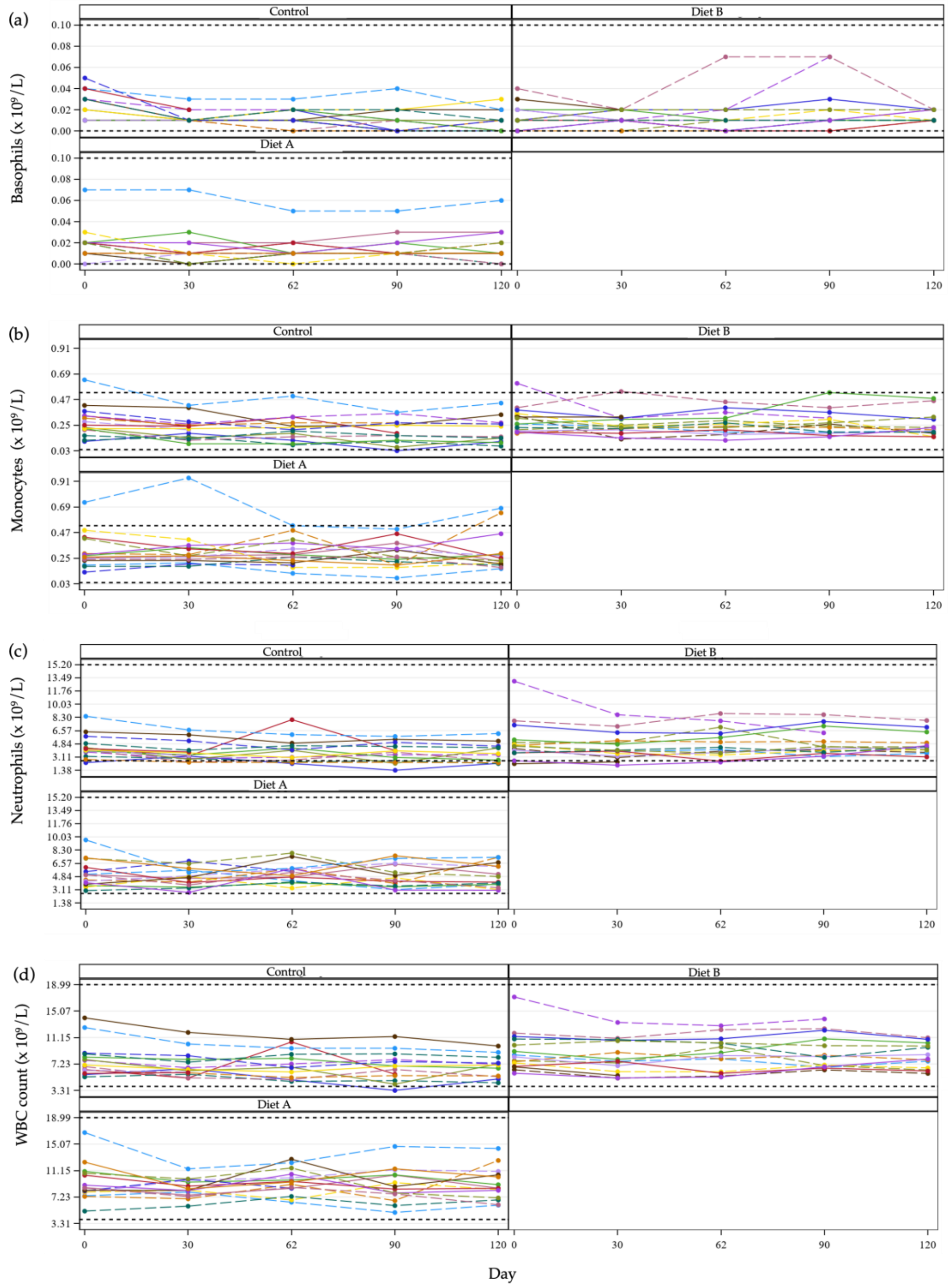

Figure S3. Linear plots of complete blood count parameters for individual cats by treatment group showing (a) basophils (x 10^9/L); (b) monocytes (x 10^9/L); (c) neutrophils (x 10^9/L); (d) white blood cell (WBC) count (x 10^9/L) at days 0, 30, 62, 90 and 120. These parameters showed significant differences in pairwise group mean comparisons between Diet A and Diet B groups compared with control. - - - - - - - - - denotes reference ranges.

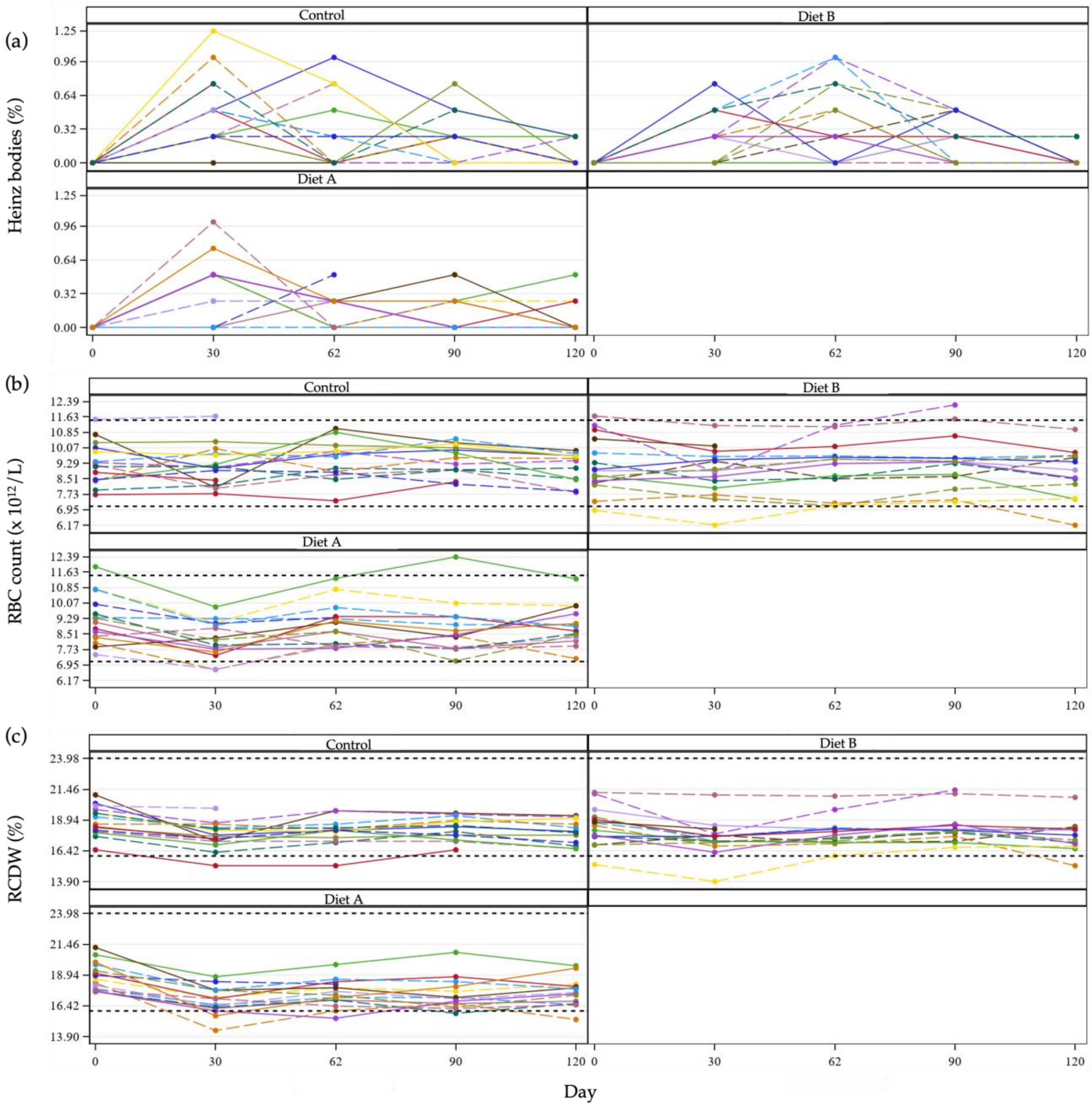

Figure S4. Linear plots of complete blood count parameters for individual cats by treatment group showing (a) Heinz bodies (%); (b) RBC (red blood cell) count (%); (c) RCDW (red cell distribution width) (%) at days 0, 30, 62, 90 and 120. These parameters showed significant differences in pairwise group mean comparisons between Diet A and control groups. - - - - - - - - - denotes reference ranges. Note that there is no reference range for Heinz bodies as they are expected to be present at very low levels in healthy cats.
